# Cytokines in the Cerebrospinal Fluids of Patients with Chronic Fatigue Syndrome/Myalgic Encephalomyelitis

**DOI:** 10.1155/2015/929720

**Published:** 2015-03-05

**Authors:** D. Peterson, E. W. Brenu, G. Gottschalk, S. Ramos, T. Nguyen, D. Staines, S. Marshall-Gradisnik

**Affiliations:** ^1^Simmaron Research, 948 Incline Way, Incline Village, NV 89451, USA; ^2^Griffith Health Institute, School of Medial Sciences, National Centre for Neuroimmunology and Emerging Diseases, Griffith University, Parklands, QLD 4222, Australia

## Abstract

*Objectives*. Previous research has provided evidence for dysregulation in peripheral cytokines in patients with Chronic Fatigue Syndrome/Myalgic Encephalomyelitis (CFS/ME). To date only one study has examined cytokines in cerebrospinal fluid (CSF) samples of CFS/ME patients. The purpose of this pilot study was to examine the role of cytokines in CSF of CFS/ME patients. *Methods*. CSF was collected from 18 CFS/ME patients and 5 healthy controls. The CSF samples were examined for the expression of 27 cytokines (interleukin- (IL-) 1*β*, IL-1ra, IL-2, IL-4, IL-6, IL-7, IL-8, IL-9, IL-10, IL-12p70, IL-13, IL-15, IL-17, basic FGF, eotaxin, G-CSF, GM-CSF, IFN-*γ*, IP-10, MCP-1 (MCAF), MIP-1*α*, MIP-1*β*, PDGF-BB, RANTES, TNF-*α*, and VEGF) using the Bio-Plex Human Cytokine 27-plex Assay. *Results*. Of the 27 cytokines examined, only IL-10 was significantly reduced in the CFS/ME patients in comparison to the controls. *Conclusions*. This preliminary investigation suggests that perturbations in inflammatory cytokines in the CSF of CFS/ME patients may contribute to the neurological discrepancies observed in CFS/ME.

## 1. Introduction

Chronic Fatigue Syndrome/Myalgic Encephalomyelitis (CFS/ME) is a disorder with an uncertain prognosis due to limited interventions for management and the lack of successful treatments. Additionally, CFS/ME is known to involve impairments of various physiological systems including the immune and neurological systems [1–4]. These may manifest as cognitive impairments, including reduced memory and concentration, pain, exertional fatigue, and other symptoms [2, 5–7].

Immunological dysregulation has been proposed as a significant component of the CFS/ME pathomechanism. Reductions in natural killer (NK) cytotoxic activity and elevations in regulatory T cells (Tregs) are the most consistent findings associated with CFS/ME [8–10]. While cytokines have been investigated in CFS/ME patients, the cyclical nature of cytokine secretion makes it difficult to determine the specific cytokine(s) implicated in the pathomechanism of CFS/ME. Additionally, the results are frequently inconsistent; for example, elevations in interleukin- (IL-) 8 [5, 11] and decreases in IL-8 have both been reported [12]. In CFS/ME, cytokines such as IL-4, IL-10, IL-17, tumor necrosis factor- (TNF-) *α*, and interferon- (IFN-) *γ* have been observed to be equivocally expressed in the serum and plasma samples and following mitogenic or inflammatory stimulation of lymphocytes [12–15].

The CNS is usually regarded as an immune privileged site; hence compromise of the normal balance of cytokines, cells, and neurotransmitters in the CNS may have serious consequences [16–19]. Cells of the CNS such as microglial cells are known to secrete cytokines under certain conditions and abnormal levels of cytokines in the CNS are usually attributed to injury, infection, or other insults to the CNS [20, 21].

As CFS/ME patients have been shown to present with a number of neuroimmune abnormalities, examining the cytokine profile of the CNS may be important. Hence, the purpose of this pilot study is to examine the role of 27 cytokines in CFS/ME patients.

## 2. Methods

### 2.1. Participants

An expedited review was obtained from the Griffith University Ethical Committee (MSC/02/13/HREC) prior to the commencement of this study. The study comprised 18 CFS/ME patients and 5 healthy controls. CFS/ME patients were defined using the 1994 Centre for Disease Prevention and Control Criteria (1994 CDC) for CFS/ME. CSF samples were collected from patients in the USA at the time of diagnosis via lumbar puncture. All samples had 0-1 red blood cells on tube number 1. All samples with blood contamination were excluded. Samples were collected according to local protocols and stored at −80 degrees prior to being shipped to the National Centre for Neuroimmunology and Emerging Diseases (NCNED) for cytokine analysis. For the purpose of this project, a total volume of 200 *μ*L of CSF was used for the cytokine analysis.

### 2.2. Multiplex Cytokine Analysis

The following cytokines and chemokines, IL-1*β*, IL-1ra, IL-2, IL-4, IL-6, IL-7, IL-8, IL-9, IL-10, IL-12p70, IL-13, IL-15, IL-17, basic FGF, eotaxin, granulocyte-colony stimulating factor (G-CSF), granulocyte macrophage-colony stimulating factor (GM-CSF), IFN-*γ*, interferon gamma-induced protein-10 (IP-10), monocyte chemoattractant protein-1 (MCP-1), macrophage inflammatory protein 1 (MIP-1)*α*, MIP-1*β*, platelet-derived growth factor- (PDGF-) BB, regulated on activation, normal T cell expressed and secreted (RANTES), TNF-*α*, and vascular endothelial growth factor (VEGF), were simultaneously examined in all CSF samples from all participants via the Bio-Plex Pro Human Cytokine 27-plex Assay (Bio-Rad) as per manufacturer's instructions. Briefly 50 *μ*L of CSF samples and various concentrations of the assay standards were added in duplicates to a 96-well plate containing magnetic beads. The plate was incubated for 30 minutes following which a wash step was applied; the plate was subsequently coated with biotinylated detection antibody solution and incubated for 30 minutes. After the 30 minutes incubation, the plate was washed and streptavidin-conjugated phycoerythrin was added to the 96-well plates and incubated for 10 minutes. The plate was washed after this final incubation and assay buffer was added to each well. Data was acquired using the Bio-Plex Array Reader system 2200 (Bio-Rad) [22]. A standard curve was derived using the different concentrations of the assay standards. All CSF samples from participants were assayed on the same plate at the same time in duplicates. Intra-assay variability was represented as the coefficient of variation as per manufacturer's instructions.

### 2.3. Statistical Analysis

Statistical analysis was performed using GraphPad Prism and SPSS. All data are presented as mean ± standard error of the mean. The Mann-Whitney nonparametric test was used to determine differences in the groups while spearman coefficient was used to examine the presence of correlations among the cytokines examined. Data was considered significant where *P* value was <0.05.

## 3. Results

**Figure 1:**
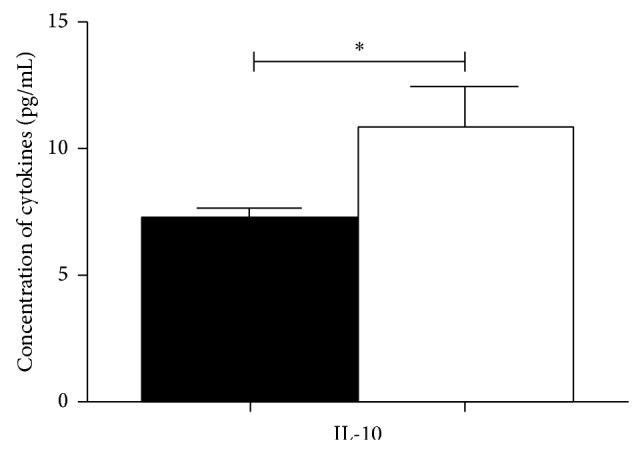
Concentration levels of IL-10 in CFS/ME patients in comparison to controls. IL-10 was the only cytokine significantly decreased in the CSF samples of the CFS/ME patients. The black bars represent data from the CFS/ME patients and the white bars represent data from the control patients. Data is represented as mean ± standard error of the mean (SEM).

Significant reductions in the concentration of cytokine IL-10 were observed in the CFS/ME patients compared with controls (Figure 1). There were no significant differences in the levels of IL-1r*α*, IL-2, IL-6, IL-7, IL-8, IL-9, IL-12p70, IL-13, IL-15, IL-17, basic FGF, eotaxin, G-CSF, GM-CSF, IFN-*γ*, IP-10, MCP-1, RANTES, TNF-*α*, and PDGF-BB between the CFS/ME group and the control group (data not shown). IL-4, IL-1*β*, MIP-1*α*, MIP-1*β*, and IL-5 were below the limit of detection.

## 4. Discussion

In the CNS, microglial cells have the capacity to secrete cytokines, act as antigen presenting cells, and induce phagocytosis [20, 23–25]. These cells may have either protective or pathological effects on the CNS function. The cytokines produced by the microglial cells include IL-4, IL-10, IL-6, IL-13, and IFN-*γ* [21].

The present pilot study has shown that cytokine IL-10 was significantly decreased in the CFS/ME patients. IL-10 is secreted by almost all cells of innate and adaptive immune system and it protects autoreactive and inflammatory reactions by dampening Th1 immune related responses. IL-10, previously described as a cytokine synthesis inhibitory factor, displays immunoregulatory as well as immunostimulatory activities, prevents autoreactivity [26] and T cell proliferation, and protects against autoimmunity [27]. Additionally IL-10 reduces B7-2 and CD28 signalling, inhibits nitric oxide secretion, degrades cytokine related mRNAs, and decreases the expression of MHC II molecules [28–30]. Importantly, IL-10 has positive and negative effects on several signalling pathways that are related to the Janus kinase/signal transducer and activator of transcription; hence, modulations in IL-10 may affect inflammatory signals and cellular process in the CNS [31]. Antigen presenting cells particularly macrophages and dendritic cells produce IL-10; this is important during inflammatory response to sepsis and infection [32]. Myeloid derived IL-10 regulates the function of T cells and the production of IL-10 by antigen presenting cells [32]. Hence, reduced levels of IL-10 may suggest compromises to the function and regulation of these cells. Microglia are the predominant myeloid cells in the brain and are known to produce IL-10 [33]. Compromises to these cells may contribute to the low levels of IL-10 observed in this pilot study.

A reduction in IL-10 may increase inflammation in the CNS as it may suggest increases in c-Jun N-terminal kinase (JNK) which is a known inducer of helper T cell differentiation and secretion of proinflammatory cytokines [34]. As hippocampal related IL-10 is known to suppress JNK, reduced levels of IL-10 may have significant implications on the inflammatory processes in the CNS. IL-10 therefore has a major anti-inflammatory role in the CNS which is required for CNS homeostasis and normal functioning. Survival of glial and neuronal cells is to some extent dependent on IL-10 as it augments neurotrophic factors [35]. Reduced levels of IL-10 may imply an increase in the synthesis of certain cytokines such as IL-1*β*, IL-8, IL-6, IL-12, and TNF-*α* [36]. However, we did not observe a substantial increase in the levels of these cytokines in the CNS; hence, other mechanisms may be acting to compensate for the reduced levels of IL-10. Only one study has reported changes in IL-10 in the CSF of CFS/ME patients, and these were increased [37]. This finding is in contrast to our present findings and this may be due to the heterogeneity of the disease, different analytical methods, and the presence of divergent patient subgroups. Nonetheless, a decrease in CNS IL-10 may be related to symptoms of fever reported in the CFS/ME cases [38] and this may be important for future investigations.

Whether or not the profile of cytokines in the CNS is similar to that in the periphery remains to be determined. Further studies are therefore required with larger samples to determine the role of CNS cytokines in CFS/ME.
